# Development of an 360-degree virtual reality video-based immersive cycle training system for physical enhancement in older adults: a feasibility study: Development of immersive virtual cycle for older adults

**DOI:** 10.1186/s12877-021-02263-1

**Published:** 2021-05-22

**Authors:** Namsu Lee, Wonjae Choi, Seungwon Lee

**Affiliations:** 1 Department of Physical Therapy, Graduate School of Sahmyook University, Seoul, Korea; 2 Department of Physical Therapy, Joongbu University, Geumsan-gun, Korea; 3 Department of Physical Therapy, Sahmyook University, Hwarangro 815, Nowon-gu, 01795 Seoul, Republic of Korea

**Keywords:** Virtual Reality Exposure Therapy, Equipment and Supplies, Dizziness

## Abstract

**Background:**

Recently, there is an increased number of studies that use 360° virtual reality (VR) video for medical and rehabilitative purposes. However, the 360° VR video experience for older adults has not yet been investigated. This study aimed to examine the validity of an 360° VR video-based immersive cycling training system (360° VRCTS) for older adults and to provide preliminary evidence of efficacy.

**Methods:**

We developed a new virtual reality training system using an immersive environment 360° VRCTS. Five healthy older adults (2 males and 3 females) participated in this study. The system was tested in a single training session (biking for 20 min while viewing a 360° VR video scene through a large curved screen) to identify its strengths and weakness. The usability and acceptability of our system were measured using the system usability scale (SUS) and the simulator sickness questionnaire (SSQ).

**Results:**

All participants successfully completed the session without any discomfort. The average score for the SUS was 94.60 (range, 90–100), indicating high usability of the technology. The average score for the SSQ was 2.24 (standard deviation = 2.05), indicating that the system is well tolerated and has few side effects.

**Conclusions:**

The 360° VRCTS may be a useful indoor training system for older adults due to its easy manipulation, high usability, and limited cybersickness.

**Trial registration number:**

Clinical Research Information Services (CRiS), KCT0003555, Registered February 25, 2019, https://cris.nih.go.kr/cris/index/index.do.

## Background

An increase in the population of older adults is associated with an increase in the prevalence of physical, psychological, and social problems. Additionally, medical expenses have increased substantially over time, putting a strain on national finances. The limited financial support for medical expenses of older adults has resulted in a paradigm shift from existing policies focused on the treatment of diseases to policies for disease prevention by promoting exercise and physical activity [1, 2].

The World Health Organization (WHO) recommends outdoor activities, such as walking and cycling, for older adults aged ≥ 65 years [3]. Indeed, outdoor activities provide physical, mental, and social advantages for individuals of all ages [4]. However, currently, it difficult to perform outdoor activities due to the lack of an adequate environment for walking or cycling resulting from is rapid urbanization. Air pollution, such as fine dust, also add to the limitations of outdoor activities [5, 6]. Furthermore, older adults need to be cautious while performing outdoor activities, since they are more vulnerable to environmentally harmful factors and more susceptible to circulatory and respiratory diseases due to air pollution compared to younger adults [7]. Therefore, despite the advantages of the outdoor environment, an alternative is needed that would allow older adults to exercise indoors.

Physical activities that can be performed indoors are, in most cases, aerobic exercises using treadmills or stationary bicycles. Stationary cycle training is advantageous in that it can be applied to frail older adults as a non-weight bearing activity, with less impact on the joints [8, 9]. However, more than half of older adults discontinue this type of exercise within six months, since they lose interest in the simple, repeated type of activity that characterized this type of exercise. Indeed, a study by Hagberg et al. (2009) reported that interest and pleasure are important factors that reinforce performing any type of exercise [10]. One way to account for these factors is virtual reality (VR).

VR is a 3D graphic-based technology that provides a realistic experience to users using 360° virtual environments (VEs) that allows them to interact with images on a screen through a variety of interfaces [11]. Immersive VR refers to a type of VR in which a screen encompasses the entire field of vision of the user to allow user to be immersed in a computer-generated environment using equipment such as head-mounted display (HMD-VR, e.g., Oculus Rift) or projection-based system (e.g., a CAVE) [12]. However, a non-immersive VR environment refers to the least interactive application in VR technology and is typically implemented in a 2D environment. Immersive VR using HMD is proposed as a more effective training method than non-immersive VR, since it provides a more realistic sensory motor experience for the user [12]. Unlike movements in the real world, exercise through VR has no temporal and spatial constraints, and since it can arouse interest in the user and provide them with an enjoyable experience, it may motivate the user to exercise more consistently [11]. VR-based cycle systems for exercise display either virtual exercise paths or actual video created in 3D graphics through an HMD, a computer automatic VE (CAVE) system, or a large front screen. During exercise, the system matches the speed of the user pedals with the rate of change of the screen. This system maximizes immersion and a sense of reality for users cycling in VR, reducing the number of shortcomings associated with conventional exercise cycles, which are monotonous [13–15]. However, most VR-based exercise cycle systems are implemented using HMDs with virtual motion paths made in 3D graphics. HMDs have the advantage of increasing the sense of reality and immersion; however, there is concern that older adults using HMDs may experience more side effects, such as cybersickness, than younger individuals, both in terms of frequency and severity [16]. Indeed, the total score of the simulator sickness questionnaire (SSQ) was lower in older adults than in younger adults [17]. Therefore, there is a need for a VR-based cycle training system that can minimize the side effects of VR and enhance physical activity in older adults.

Recently, interest in 360° VR videos has increased. Most 360° videos are made of ultra-high definition (UHD) video, 4 K UHD class or higher, to produce panoramic video in all directions (360°) with one or more cameras, typically filmed in real world locations. Unlike standard video in which the viewpoint is fixed by the person shooting the video, the 360° VR video allows for the use an input device, such as a keyboard, mouse, or accelerometer, in order to arbitrarily set the intended viewpoint [18]. As interest in 360° VR video content has increased, there has been an increasing number of studies applying 360° VR video to the field of medicine and rehabilitation [19–21]. In particular, Calogiuri et al. (2017) showed the potential of combining exercise with 360° VR video in a group performing treadmill training with 360° VR video that achieved similar physical benefits compared to a group performing outdoor walking [19].

However, since 360° VR video content still uses either stationary video or video-based content, users can only passively view content and are not able to participate through interaction, which is one of the main characteristics of VR. Furthermore, 360° VR video has not solved the problem of cybersickness, since it is provided through immersive HMDs and mobile phone-type HMDs with tracking technology to control 360° VR video. Calogiuri et al. (2017) argued that in order to utilize 360° VR video properly, side effects such as cybersickness should be resolved [19].

Therefore, this study aimed to develop a 360° VR video-based immersive cycling training system (360° VRCTS) that responds to the user’s head movement to reduce cybersickness in HMD as reported in previous studies. We hypothesized that 360° VRCTS would result in a user experience characterized by high satisfaction and low cybersickness.

## Methods

### Study design and participants

This study was designed as a feasibility study in which healthy older adults were eligible to participate. To evaluate the usability of the 360° VRCTS, this study recruited older adults residing in B city in Gyeonggi Province.

Individuals 65 ≥ years of age without previous experience with VR participated in the study. The participants had no eye diseases, such as cataracts, glaucoma, central and peripheral nerve injuries, or cardiopulmonary disease. Individuals who had previously experienced dizziness, vestibulopathy, or cyber sickness were excluded. Participants self-reported their general characteristics and medical histories. The minimum sample size required for usability studies was 5 subjects [22]. Five participants were recruited on a first-come, first-serve basis (3 men and 2 women). The mean age of the participants was 69 years (SD = 3.67). Convenience sampling was performed in this study.

 The study was approved by the Institutional Review Board of Sahmyook University. The trial was registered with the International Clinical Trials Registry Platform (KCT0003555). All participants were provided with an explanation of the purpose and risk of the study prior to preparation of the consent form, and written informed consent was obtained based on the revised Declaration of Helsinki (2013).

### 360° virtual reality video-based immersive cycling training system

To develop cycle content based on the physical, psychological, and mental characteristics of older adults, the content must be designed to have a realistic background composition and easy-to-use controls to enable the user to have visual immersion. The 360° VRCTS consists of a visual display, cycle interface, and immersive interface that can provide real-time visual feedback.

All data were processed in Arduino (Arduino Micro, Arduino, Italy) and sent to Unity 3D (Unity 5, Unity Technologies, San Francisco, USA). The laptop allow manual pedaling speed using magnets placed on the pedal arm through the reed switch sensors. The MPU6050 sensor was mounted on a Bluetooth headset to recognize the movement of the user’s head and set the camera’s movement within the sphere, which was implemented in Unity.

**Fig. 1:**
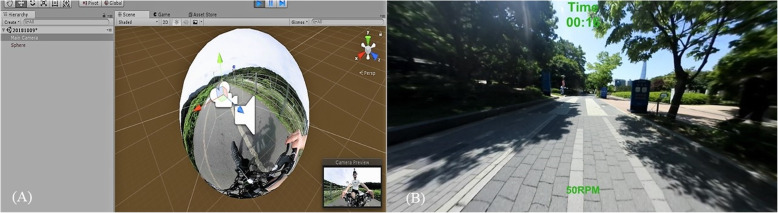
The 360° virtual video environment. **a** Insert 360° image into Unity 5 engine **b** Screenshot of actual image projected onto display

The visual display of the cycleway was created using the Unity 5 engine. Figure 1 shows an overview of the screenshot of the cycleway. Assembling a 360° VR video requires capturing images of the real environment to populate the VE. The 360° VRCTS consisted of a 360° video reproducing riding cycle. A wide field of view (FOV) integrates surrounding visual signals, improving perception of self-motion and immersion. The ideal FOV was between 80° and 200° [23]. The FOV in the 360° VRCTS environment was 110°. The video was filmed in a familiar real world surrounding, such as bike paths, three months before the start of the study using a camera (Nikon KeyMission 360, Nikon, Tokyo, Japan) and was adjusted for viewing in Adobe (Premiere Pro CC 2018, Adobe, San Jose, USA). The audio was recorded simultaneously to capture sounds such as cycle pedals, passing people’s voices, and other natural phenomena.

**Fig. 2:**
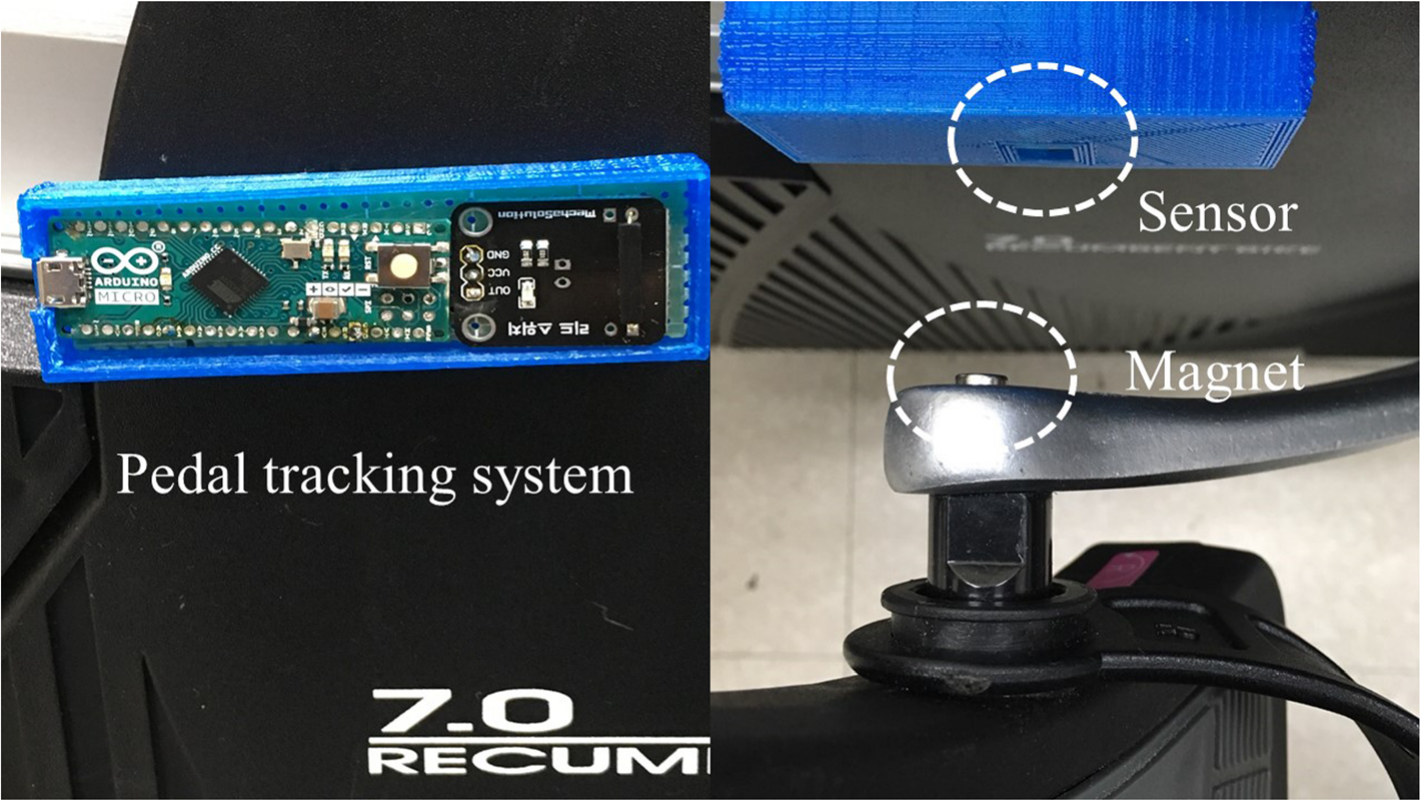
Reed switch sensor and magnet on manuped chassis

The cycle interface portion of the setup consisted of a stationary recumbent cycle (New Balance 7.0r Recumbent) on which cycling was tracked as cadence, by a mechanism in which a small magnet attached to a pedal arm crossed a Hall effect sensor placed on the chassis. Every time the magnet crosses the sensor, the sensor triggers and outputs a signal, which signifies a complete rotation cycle of the pedal arm (Fig. 2). Similar tracking systems have been used in cycle computers for decades, in which cadence was used to calculate speed. These system remain the standard of many ANT-based systems developed by Garmin [13]. The Hall effect sensor was connected to an Arduino microcontroller, which was connected to a personal computer running the VE. The Arduino code streamed the value read by the Hall effect sensor to the personal computer via USB, sending a continuous sequence of revolutions per minute (RPM) values to Unity 3D. In this way, the data from the sensor applied to the cycle were routed to a Unity 3D script that controlled the speed of movement in the VE.

**Fig. 3:**
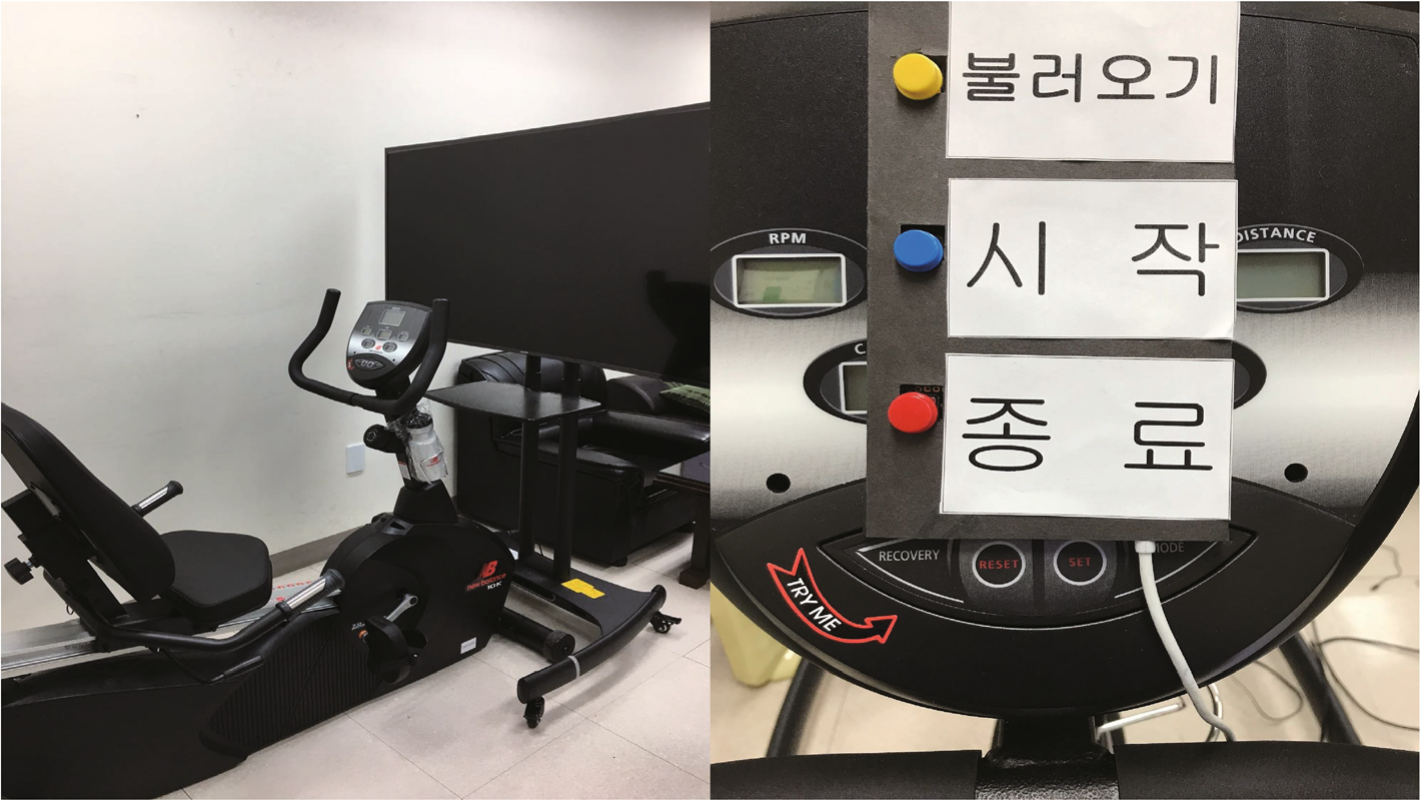
The stationary recumbent bike and interface used in the experiment: **a** 불러오기 (Load), **b** 시작 (Start), and **c** 종료 (End)

The latest immersive devices available on the market provide many features using a variety of technologies; however, they are not easy for older adults to use. For older adults, the function of the hardware interface should be minimized and designed to be easily handled by a user. Taking this into account, a control device using Arduino Micro was fabricated and attached to a stationary recumbent cycle so that the content could be run by simple selection (Fig. 3).

To provide maximum immersion and sense of reality, we matched the 360° cycle video speed with the stationary recumbent cycle speed whenever possible. Using a magnetic sensor attached to the main body of the stationary recumbent cycle, the RPM based on the pedal rotation was acquired as the speed signal data by the switch. The 360° video playback speed was adjusted by substituting the speed data measured by the stationary recumbent cycle pedals into the Unity video playback speed (e.g., 30 RPM = 0.5 video playback speed, 40 RPM = 0.8, 50 RPM = 1.0, etc.). Speed was defined by calculating the contact signal generation period between the disk that rotated when the cycle pedal was moved and the magnetic sensor attached to the cycle body and combining this with the running distance per unit time (2π * radius of wheel). Exercise time and RPM were displayed on the screen to encourage subjects to participate in the exercise (Fig. 1).

**Fig. 4:**
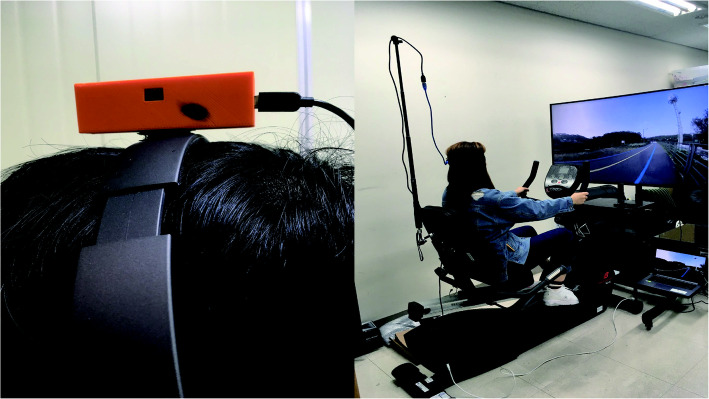
MPU6050 sensor-based head tracking system and experimental setup

In a typical TV environment, as in the case of an HMD and other mobile devices, there is the physical constraint, in which the display itself cannot be moved or change the location based on the user’s viewpoint during movement. Therefore, to implement the interface function between the TV environment and 360° VR video content, a head-tracking system was created to show the 360° VR VE based on the direction of the user’s head (Fig. 4). This was fabricated based on the MPU6050 inertial sensor and attached to a Bluetooth stereo headset for use.

### Procedure

The experimental equipment consisted of a 55” UHD curved screen TV (Samsung 7350, Samsung, Seoul, Korea) stationary recumbent seating cycle (7.0r Recumbent, New Balance, Taipei, Taiwan), Arduino Micro (ATmega32U4) controller, laptop (VivoBook X510U, ASUS, Taipei, Taiwan), and Bluetooth stereo headset (Beatles M-15, Unicorn, Seoul, Korea).

All participants fasted for 2 h before the experiment to distinguish discomfort caused by post-meal exercise and cybersickness. The laboratory temperature was maintained at 24 °C, and exercise was performed under low light to facilitate focus on the screen video. After the researcher’s demonstration, participants were asked to perform all the experimental procedures. The participants performed non-VR warm-up cycles for 3 min before the start of the experiment. During the warm-up exercises, the researchers explained and demonstrated how and when the 360° VRCTS began and ended, and its inherent characteristics. Participants were told to watch the video by moving it up/down and left/right, since it was a 360° video. While freely cycling for 20 min, the participants experienced a 360° VR video-based cycle system, in which the video speed varied depending on the cycle speed and, although it was a video, the screen viewpoint changed when it was moved up/down or left/right. If participants felt excessive eye fatigue, repulsion, or cybersickness during the cycle, they were instructed to raise their hand at any time to indicate their intention to quit or press the end button on the 360° VRCTS to exit. A researcher was always positioned next to the participant to ensure a safe experience.

At the end of the 360° VRCTS session, the researcher helped the participant end the recumbent seating cycle and led them to the room next to the laboratory. Participants rested until they fully recovered, and then filled out a questionnaire provided by another researcher.

### Outcome measures

In this study, usability is the extent to which a user experiences the developed VR system to accomplish quantified goals satisfactorily, safely, and efficiently within a well-constructed context [24]. We assessed the usability of the test system using the system usability scale (SUS), a validated measure of learnability and user satisfaction. The SUS was designed to measure the end-of-test subjective assessment of the usability of interface technologies. This survey has excellent reliability (0.85) and has been broadly used to evaluate product usability [25, 26]. Levels of agreement with 10 statements were scored using a five-point Likert scale anchored with ‘strongly disagree’ and ‘strongly agree’. The 10 questions were interrelated and used for a comprehensive evaluation of the product. The higher the SUS score, the better the product usability. Scores above 70 are acceptable, and highly usable products have score above 90. Scores below 50 indicate unacceptably low usability levels [25].

Cybersickness is a side effect felt after experiencing VR and its symptoms include nausea and dizziness [27]. The stimulator sickness questionnaire (SSQ) was used as a subjective measure to evaluate cybersickness. The SSQ consists of 16 four-point Likert scales (none, slight, moderate, or severe), such as eye fatigue, dizziness, and headache. Generally, the sum of SSQ represents the total sickness score, and nausea, disorientation, and oculomotor symptoms were calculated in the SSQ sub-item. The SSQ has been shown to be reliable in healthy adults (0.80). These factors were not completely independent. Approximately five items were evaluated for one or more factors. For example, the score for focal difficulty was used to assess oculomotor disorders and disorientation. To obtain the score of each factor, the items of each factor were added and multiplied by a specific weight. A higher SSQ score indicated that the user had more severe cybersickness. A score ≥ 20 indicates that the user experienced significant discomfort [27, 28].

### Statistical analysis

All data were recorded on a Microsoft 2016 Excel Spreadsheet and descriptive statistics were used to calculate the mean and standard deviation (M ± SD) of clinical and demographic variables. The SUS scores were calculated using the Brooke (1996) method. Based on the method proposed by Kennedy et al. (1993), SSQ scores were derived from the overall symptom value (TS: total score) and the values of three symptom subgroups (N: nausea, O: oculomotor symptoms, and D: disorientation).

## Results

**Fig. 5:**
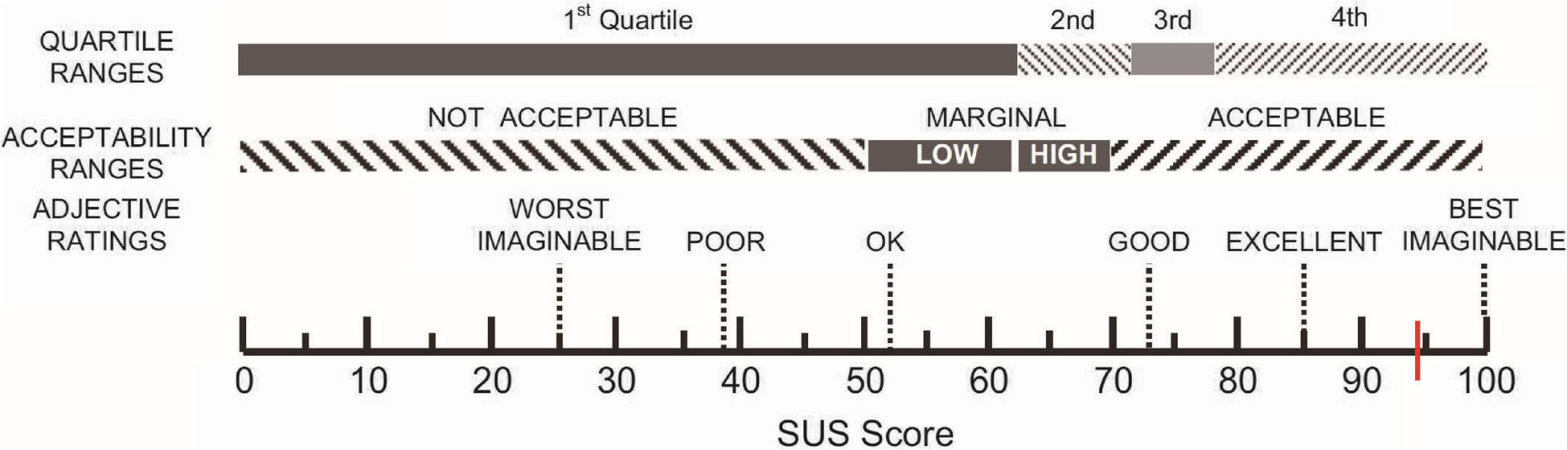
A graphic representation of the SUS score

Patient demographics and raw SUS and SSQ scores are shown in Table 1. The mean usability value, assessed with the SUS, was 94.60 (SD = 3.85) points, as shown by the red line in Fig. 5. SSQ proportional scores for nausea, oculomotor symptoms, and disorientation were 3.82 (SD = 5.23), 0.00, and 2.78 (SD = 6.23) points, respectively. Among the three subgroup scores, the SSQ proportional score for oculomotor symptoms (0.00) was the lowest. Total simulator sickness scores were 2.24 (SD = 3.85) points. All participants were able to complete the study without exceeding the lowest or low levels of simulator sickness symptoms.

**Table 1:** Participant demographics, usability, and cybersickness

ID	Sex	Age (range, years)	Weight (kg)	Height (cm)	SUS (points)	SSQ-N (points)	SSQ-O (points)	SSQ-D (points)	SSQ-TS (points)
1	Male	65–74	61	163	95.70	0.00	0.00	0.00	0.00
2	Female		48	155	95.00	0.00	0.00	13.92	3.74
3	Female		46	149	92.50	0.00	0.00	0.00	0.00
4	Female		66	147	90.00	9.54	0.00	0.00	3.74
5	Male		67	165	100.00	9.54	0.00	0.00	3.74
Mean (SD)		69.00 (3.85)	57.60 (9.96)	155.80 (8.07)	94.60 (3.85)	3.82 (5.23)	0.00 (0.00)	2.78 (6.23)	2.24 (2.05)

*SUS *system usability scale, *SSQ* simulator sickness questionnaire, *N* nausea, *O* oculomotor, *D* disorientation, *TS* total score

## Discussion

This feasibility study was aimed at investigating the usability and stability of an immersive cycle training system based on 360° VR video, which was developed to improve indoor physical activity for older adults aged ≥ 65 years. The results of our study are encouraging. SUS scores (94.60 ± 3.85) were very high, indicating high levels of acceptability, ease of use, learnability, and confidence when using the system. Cybersickness is one of the most common side effects in VR applications, but we found that our 360° VRCTS elicited only minor symptoms of nausea (3.82 ± 5.34) or disorientation (2.24 ± 3.85).

In previous studies [29–33], VR cycling was used for older adults or patients with stroke as a method of exercise rehabilitation, with improvements in aerobic capacity, walking endurance, postural balance, adherence, and executive function. However, to date, no research has been conducted on 360° VR video. Older adults are known to have a negative propensity for new technologies [34, 35]. Since the use of new technology depends on the user’s attitude [36], it is important to determine older adults’ attitudes toward the content. Attitudes were measured with SUS, which included questions measuring ease of use, usefulness, and intent to use 360° VRCTS. As a result of this study, 360° VRCTS has been shown to be excellent regarding usability by older adults. An SUS score higher than 70 is regarded as acceptable usability, and scores higher than 85 are regarded as excellent usability. If the score is higher than 90, it is considered to be truly excellent [25]. The mean SUS score in this pilot study was 94.60 ± 3.85 (range 90–100), which indicate a satisfactory level of usability. We found that adaptation of our system was not necessary.

Despite VR advantages, more than 60 % of users experience cybersickness when encountering VR, which can lead to unpleasant experiences for the user and can reduce the duration of VR use [37]. One study compared cybersickness levels experienced with HMD, desktop-computer, and reality theaters in healthy young adults [38]. The results showed that experiencing VR using HMDs caused more cybersickness symptoms than experiencing VR using non-immersive devices, such as desktops or projectors. While HMDs have the advantage of increasing the sense of reality and immersion, there is a concern that older adults are more likely to have serious side effects in their immersive experience with HMDs than younger individuals [16, 39]. Therefore, we used a projection-based system based on a curved TV to minimize cybersickness symptoms in older adults aged ≥ 65 years.

Analysis of the SSQ total score showed that the 360° VRCTS developed by the authors did not cause cybersickness or the effect was minimal. Examination of the subscores (N, O, D) showed that two participants, one male and one female, had nausea symptoms (sweating), and another female participant had disorientation symptoms (pressure felt in the head), but all symptoms were insignificant (slight). The head pressure felt by one of the participants was due to use of the headphones. Indeed, this participant did not report any symptoms in the other subgroups (N, O), so it is thought that the felt head pressure may indeed have been caused by headset-wearing problems rather than disorientation caused by the 360° VRCTS. A previous study examined differences in the user’s VR experience based on HMD weight [40]. Participants experienced VR with an HMD (Sony Glasstron LDI-D100B) weighing 340 or 490 g. Users felt more pressure on their nose when wearing a heavier HMD; therefore, they experienced discomfort independent of the occurrence of cybersickness. These findings support our argument that a headset-wearing problem caused head pressure symptoms in the disorientation category.

It is noteworthy that our study showed no oculomotor symptoms in any of the participants. Cybersickness, unlike common motion sickness, is mainly caused by vision [41–44]. According to the rest frame theory on causes of cybersickness, our nervous system efficiently calculates visual movement and recognizes one rest frame for spatial awareness. A rest frame is a reference frame designed to recognize our current position, direction, and movement in space and to make spatial judgments. In a VE, different rest frames are recognized by different motion cues, and cybersickness occurs due to interference in the nervous system between the different rest frames determined by these motion cues [42, 45, 46]. Previous studies that aimed to reduce cybersickness based on the rest frame theory added a visual stimulus that was always fixed and was independent of movement of the VR content. For example, one can fix a cloud or tree in the same location, independent of the motion of the content [47], or render a frame on the front or back of the screen [42]. We examined whether these video elements could alleviate the cybersickness symptoms of the user. When a fixed visual stimulus was added, the users’ cybersickness symptoms were significantly reduced, regardless of the brightness of the frame pattern [42]. Previous researchers interpreted that the various types of fixed visual stimuli mentioned above served as a frame of reference to help users pinpoint their position in VR.

This study included multiple efforts to reduce cybersickness. First, visual elements fixed in 360° VRCTS videos were inserted based on the rest frame theory. At the start of the video, the environment seen from the front of the cycle was set as the user’s point of view, with the play time displayed at the top of the screen and the RPM speed at the bottom. Even if the user turned his/her head, the play time and RPM speed were fixed on the front of the screen so that the user could recognize the front of the screen to stabilize the point of view during the video. Another way we attempted to reduced cybersickness was making it so that our content only allowed a single rotational axis movement whenever user tried to move on the 360° video screen by moving his/her head. For the y-axis movement, the screen moved only left and right by blocking the z-axis and x-axis movements. When the x-axis movement occurred, the screen moved only up and down by preventing movement of the y-axis and the z-axis.

The purpose and content of VR can be divided into active and passive experience types. In previous studies, users who consistently experienced passive experiences reported more severe cybersickness [48–50]. These results indicate that cybersickness symptoms can be alleviated when the user has control over the VE and when them can predict changes in the situation based on their behavior. The 360° video content currently available typically allows passive viewing. Based on previous studies, the third method that we used to reduce cybersickness was to provide active experience. Unlike previous studies that provided only 360° video using HMD [51, 52], we provided an optical flow effect in which the pedal speed (measured by the cycle-ergometer in RPM) and the video speed were linked to allow the user to actively participate.

Owing to the nature of VR technology, the most direct way to realize a high-fidelity environment is to maximize the visual reality of the video by using the best graphic technology. To create a high-immersion VR experience for the user, the developers tried to implement a VR that was very similar to the real world. However, despite the expectation that cybersickness occurrence would decrease as VR becomes more similar to the real world, cybersickness symptoms have been shown to be exacerbated by the addition of texture detail to increase the graphic sense of reality [41, 53], and users who experience complex 3D background configurations show high SSQ scores [54, 55]. High-fidelity content implemented at the current level of technology can still be unrefined from the perspective of human cognitive systems, and VEs that mimic the real world to enhance the faithful representation of VR (e.g. virtually constructed buildings, trees, and other structures) may, in fact, induce cybersickness in the user by highlighting the difference between VR and reality. Therefore, as a fourth method to encourage immersion in VR and prevent cybersickness, this study captured video of an actual cycle road through a 360° video camera and constructed the VR environment from the actual video.

Finally, we did not use an HMD, which is a display method used by existing VR systems, or a CAVE system that uses multiple monitors or large screens, instead we used a curved TV, which is a new method for displaying VR. In other words, the shape of the screen widens the FOV, and the user experiences an improved sense of depth. This effect results in optimum immersion for the user. In a previous study on the occurrence of cybersickness caused by a specific display device, serious cybersickness occurred not only with an HMD, but also with a large flat-panel screen [56]. Unlike an HMD, where the user’s FOV is completely blocked and the user experiences only VR, a large flat-panel screen allows the user to observe the surrounding environment in addition to the content. Therefore, the user experiences greater inconsistencies between visual stimuli when they experience VR on a screen (the content of the VR moves in many directions, while the visual stimulus of the external environment is fixed in one direction), resulting in cybersickness. To solve this problem, previous researchers conducted experiments that allowed only a limited viewing range (32° × 17°) on large screens and reduced the size of the content, and they observed that cybersickness was significantly reduced [56]. Therefore, in this study, a curved TV was used to provide the optimum viewing range of the content while minimizing the range of the user’s observation of the external environment. The 55” curved TV we used had a curved monitor radius of 4,200 R and provided an FOV of 28.42°.

### Limitations

Currently, a widely used method to measure cybersickness is the questionnaire, which relies on subjective reporting by individuals. The questionnaire measurement has the advantage that it is intuitive, and anyone can easily complete it. However, since cybersickness symptoms are collected from the user at the end of the VR experience, adverse reactions cannot be collected in real time. There are also the issues regarding differences in the definition of cybersickness among different individuals. The questionnaire used in this study was the SSQ [28]. However, the SSQ is unable to record simple, immediate body reactions, since it has numerous questions and considers many aspects of cybersickness symptoms. In addition, a study found that participants overestimated their cybersickness symptoms when using the SSQ questionnaire [57]. This means that the questionnaire style of response collection may negatively distort the VR experience. In this study, interviews were conducted to prevent such problems, but the questionnaire method has a limitation in that it can negatively distort the VR experience. In future studies, objective measures should be used that can quantify changes in cybersickness in real time to overcome the inherent issues of subjective measures and ensure accurate results.

## Conclusions

This study is based on the development of a method that provides immersive content for cycle training based on 360° VR video, and attempts to increase interest to motivate older adults to perform indoor exercise. Although the study consisted of only five older adults (3 male, 2 female), the results were very encouraging. Participants were very motivated to use the 360° cycle content, and it did not cause cybersickness, the most common obstacle to using VR in an older adult population. In addition, there were no major difficulties for older adults when using the system and no special training was required, resulting in very high usability.

Based on these results, we believe that our 360° VRCTS can be safely used as an indoor exercise tool for older adults. Future research should focus on developing various exercise protocols using 360° VRCTS and verifying their effects using a larger number of participants. Furthermore, in combination with wearable devices that have recently attracted attention in the healthcare market, we believe that even more advanced 360° cycle content could be provided in the future.

## Data Availability

Data are available from the corresponding author upon reasonable request.

## Abbreviations

WHO: World Health Organization
VR: Virtual reality
3D: 3 Dimension
VE: Virtual environments
HMD: Head-mounted display
CAVE: Computer automatic virtual environment
UHD: Ultra-high definition
360º VRCTS: 360 degrees virtual reality video-based immersive cycling training
FOV: Field of view
RPM: Revolutions per minute
SUS: System usability scale
SSQ: Simulator sickness questionnaire
